# Possible Effects of Bright Light Therapy on Electroencephalogram-Vigilance in the Treatment of Depression in Adolescents: A Pilot Study

**DOI:** 10.3389/fpsyt.2022.820090

**Published:** 2022-05-12

**Authors:** Christoph Berger, Alexander Dück, Stephanie Gest, Lena Jonas, Michael Kölch, Franziska Martin, Olaf Reis, Jennifer Schroth, Tanja Legenbauer, Martin Holtmann

**Affiliations:** ^1^Department for Child and Adolescent Psychiatry and Neurology, University Medical Centre Rostock, Rostock, Germany; ^2^Landschaftsverband Westfalen-Lippe (LWL) University Hospital Hamm for Child and Adolescent Psychiatry, Ruhr University Bochum, Bochum, Germany; ^3^Department for Child and Adolescent Psychiatry and Psychotherapy, Brandenburg Medical School Theodor Fontane, Brandenburg an der Havel, Germany

**Keywords:** vigilance, depression, bright-light-therapy, BLT, VIGALL

## Abstract

**Background:**

Chronotherapeutic treatments for depression, such as bright light therapy (BLT), are non-invasive and produce almost no side effects. However, study evidence for reliable neurobiological changes associated with treatment response is still rare. Several studies using EEG-vigilance indicate higher arousal and a later decline during resting state in adult depressive patients compared to healthy controls. To our knowledge, there are no study reports on EEG-vigilance in depressive youth to date.

**Methods:**

A total of 11 adolescents with depression receiving BLT were compared to 11 age and gender-matched patients with depression receiving treatment as usual (TAU). The BLT was administered in the morning for 2 weeks on five consecutive days per week. The depressive symptomatology was assessed using the Beck Depression Inventory (BDI-II) and the resting state electroencephalogram (EEG) of 20 min was recorded. EEG and BDI-II were assessed before and after 10 days of treatment. Vigilance level and vigilance decline were estimated using the VIGALL toolbox.

**Results:**

Brain arousal increased after 10 days of bright light therapy in adolescents with depression. Severe depressive symptoms were associated with higher brain arousal levels; the BDI-II sum score correlated negatively with the amount of drowsiness.

**Limitations:**

The sample size was small and participants’ brain arousal at baseline was not matched and differed between BLT and TAU groups.

**Conclusion:**

The BLT might have an additional effect on brain arousal. EEG-vigilance seems to be a reliable and valid marker for neurobiological changes that are probably associated with depression and its treatment and, therefore, might be of clinical relevance.

## Introduction

Depression is a major health problem that arises during adolescence with increasing incidence rates (1, 2). Prevalence rates of depression in youth range between 5 and 6%, with a lifetime prevalence rate of 20% by late adolescence (3). The risk of suicide might be increased by 30 times (4). Despite extensive research efforts on cognitive-behavioral approaches and medication, treatment outcomes for depressive youth remain unsatisfying (5, 6). Hence, better treatment is needed. Chronotherapeutic treatments, such as bright light therapy (BLT), are non-invasive and produce almost no side effects (7). Preliminary evidence shows its feasibility not only in adults [e.g., (8)], but also in adolescents with depression [e.g., (9–12)].

However, responses to treatment mostly rely on self-reports, which underlie possible bias and lack of elucidated neurobiological changes, and are perhaps associated with treatment response. Accordingly, there has been a call for more objective markers, such as EEG biomarkers, to estimate treatment responses. However, it is unclear which EEG biomarkers should be used when depression treatment outcome is of interest (13).

Hereby, the concept of vigilance may be of help: several studies indicate higher arousal and a later decline during a resting state EEG-session in adult depressive patients compared to healthy controls (14) as well as a more stable vigilance regulation pattern in adult depressive patients compared to healthy controls (15). Hegerl and Hensch (16) discussed the avoidance of stimulation as a reaction to a tonic increased vigilance in depression in adults. To our knowledge, there are no study reports on EEG-vigilance in depressive youth to date.

The BLT in the morning stabilizes the circadian rhythm and therefore probably also changes brain arousal. BLT can decrease daytime sleepiness and induces a forward shift of sleep onset when it is carried out about 7.5 up to 9.5 h after the secretion of melatonin, referred to as dim light melatonin onset (DLMO) (17).

To summarize, vigilance can be seen as directly related to the negatively biased and increased default mode network (DMN) activity in depression, which expresses itself as rumination, resulting in increased brain arousal, perhaps, changed by BLT. Up to date, no study investigated EEG vigilance in depressed adolescents. The study aimed to assess vigilance before and after BLT for depressive adolescent inpatients, compared to usual treatment for depression [treatment as usual (TAU)].

## Methods

### Procedure

All patients admitted to the LWL university hospital Hamm between 2013 and 2014, with a clinically diagnosed depression based on the International Statistical Classification of Diseases and Related Health Problems (ICD-10) and a BDI-II (18), indicating moderate to severe depression, were screened for in- and exclusion criteria of a larger study described elsewhere (COWALI) (19). A positive vote from the ethics committee of the Ruhr-University Bochum, Germany (Registry number: 4,418–12) was obtained. Patients and their legal guards/parents signed informed consent for taking part in the study and being investigated with an EEG. A pre-test BDI-II and an EEG have been recorded non-more than 3 days before treatment (details see below). The recording started with controlled eye movements, the Berger test, and a mental calculation. Then, a resting EEG was recorded for 20 min. On the last day of 10 days of BLT or TAU, a post-EEG assessment and a second assessment *via* BDI-II were conducted similar to pre-test conditions.

### Participants

Twenty-two female patients aged between 13 and 18 years were included in the study. The two treatment groups were recruited consecutively, after the BLT group recruitment (*n* = 11) age-matched patients for the TAU group (*n* = 11) were recruited. Participants were not matched for EEG parameters. Exclusion criteria comprised psychotic symptoms, suicidality, organic brain damage, anorexia nervosa, intake of neuroleptics or beta-blockers, and an IQ of < 85. Antidepressant medication had to be stable for at least 4 weeks. Two patients in the BLT group and one patient in the TAU group dropped out for similar reasons, while 9 patients in the BLT group and 10 patients in the TAU group were finally included in the analysis.

### Treatment

Patients received BLT starting 14.56 days after admission (*SD* = 6.10 days). The BLT comprised ten sessions of 45 min with 10,000 Lux. The BLT was administered in the morning for 2 weeks on five consecutive days per week. BLT started 8.5 h after melatonin-onset, which was calculated with the help of the Morningness-Eveningness-Questionnaire (MEQ) (20). The MEQ is a self-report questionnaire, which can determine the individual chronotype. During weekends, no BLT was administered.

The TAU is based on a multimodal therapy approach including individual and group psychotherapy, as well as all kinds of care and therapy, in-hospital schooling, and family-focused interventions are usually provided.

### Electroencephalogram Measurement and Preprocessing

The resting-state electroencephalogram (EEG) of 20 min was recorded according to the 10/20 system (Fp1, Fp2, F3, F4, C3, C4, P3, P4, O1, O2, F7, F8, CP1, CP2, CP5, CP6, Fz, Cz, Pz, FC5, FC6, T7, T8, FC1, FC2, EOG, and ECG). The sampling rate was set to 200 Hz. Offline, EEG was preprocessed with BrainVision Analyzer (BrainProducts, Gilching, Germany) according to the manual of the VIGALL toolbox,^1^ which included the following steps: Filtering with Butterworth’s zero-phase filter (0.5–70 Hz, notch at 50 Hz), creating 1-s segments, rough artifact screening by visual inspection, execution of independent component analysis (ICA) and exclusion of ICA components reflecting continuous artifacts like blinks, eye movements or cardioballistic artifacts, and marking of remaining artifacts. EEG data were screened for sleep-indicating graphoelements (sleep-spindles, K-complexes), which did not occur.

### Vigilance

To keep data comparable to prior EEG vigilance research (21, 22), we used similar methods for arousal analysis. Consecutive segments of 1-s length were classified into 6 different EEG-vigilance stages: 0, A1, A2, A3, B, and B2/3 (C was not observed), ranging from wakefulness to drowsiness by using the add-on VIGALL 2.1 for BrainVision Analyzer. To do so, the VIGALL algorithm uses source localization in different frequency bands with LORETA. Further information about VIGALL, which is licensed under GPL3 is available at https://github.com/danielboettger/VIGALL or see text FOOTNOTE 1. The arousal stability index was calculated based on 1-min intervals (interval 1, segments 1–60; interval 2, segments 2–61; etc.) (For scoring criteria see Table 1). Furthermore, we calculated the mean arousal level from the whole EEG acquisition period and the relative EEG vigilance stages occurrence (number of segments of one stage/number of all artifact-free segments). Vigilance stage C of sleep onset was not observed.

**TABLE 1:** Scoring criteria of the arousal stability index.

Scoring criteria	Score
> 2/3 of all segments classified as 0 or A1	8
≥ 2/3 of all segments classified as 0 or A1,A2,A3	7
≥ 1/3 of last 10 min classified as B1	6
≥ 1/3 of second 10 min classified as B1	5
≥ 1/3 of first 10 min classified as B1	4
≥ 1/3 of last 10 min classified as B2/3	3
≥ 1/3 of second 10 min classified as B2/3	2
≥ 1/3 of first 10 min classified as B2/3	1

*The arousal stability index quantifies the extent of the arousal regulation. This index was developed by the VIGALL research group (22), lower values mean earlier arousal decline.*

### Statistical Analysis

**FIGURE 1:**
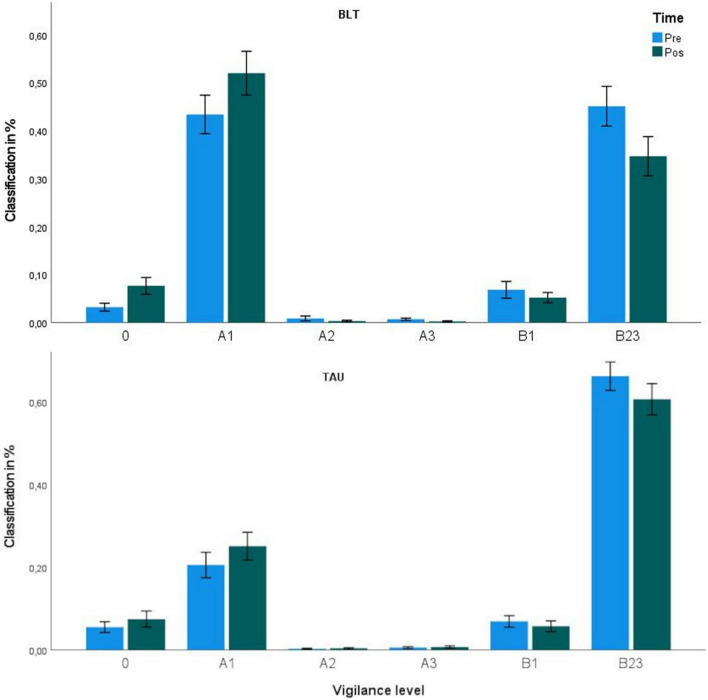
Vigilance classification distribution. BLT, Bright Light Therapy; shown are the mean percentage of vigilance level classifications. The 20 min resting-state EEG was second-wise classified by the VIGALL algorithm.

Of all vigilance classification stages, we only used A1, B23, and mean vigilance as dependent variables, because A1 and B23 were the most classified stages and can, therefore, be treated as most relevant (Figure 1). The effects of time on A1, B23, and mean vigilance were normally distributed and were tested with paired *t*-tests. For the same dependent variables, repeated measures ANOVAs were additionally modeled with the grouping factor, the inner subject factor time (pre and post), and the interaction of both. Baseline measures of vigilance were compared between the groups with a test of equivalence, the minimal detectable effect size for the two-sample *t*-test was used as the smallest effect size of interest (SESOI, d = 1.366, N1 = 9, N2 = 10, power = 0.8, and alpha error probability = 0.05). Baseline variables were seen as equivalent if confidence interval boundaries of effect sizes were smaller than the SESOI. Effects of the group-factor on the distribution of pre-post difference of arousal stability indices were analyzed with crosstabs and χ^2^-tests; the effect of time was tested with the Wilcoxon test, separately for the BLT and TAU group. We used Pearson’s correlation index to analyze the association between the BDI-II sum score and vigilance classification B23 separately for pre and post-measures. Additionally, we tested with Pearson’s correlation index whether changes in vigilance level were associated with baseline scores or with changes in the BDI-II sum score.

For the paired *t*-tests and the repeated measures ANOVA we calculated the minimum of the detectable effect size. For the two-tailed paired *t*-tests with total sample size = 18 (BLT) and 20 (TAU), alpha error probability = 0.05, and power = 0.8 resulting in minimum Cohen’s d = 0.7 (BLT) and d = 0.66 (BLT) (medium to large effect). Repeated measures ANOVA, with total sample size = 36, alpha error probability = 0.05, power = 0.8, and correlation among replication measures = 0.5, resulting in minimum Cohen’s d = 0.295 (small effect) for within factor, d = 0.961 (large effect) for between factor, and d = 0.48 (medium effect) for between and within factor interaction.

## Results

### Baseline Characteristics

The groups neither differed regarding age [*t*(17) = 0.021; *p* = 0.983; *M* = 15.61 years *SD* = 1.19] nor BDI-II sum score at admission [*t*(17) = 0.378; *p* = 0.710; *M* = 31.53 *SD* = 10.98]. Three patients under BLT got antidepressant medication (quetiapine or SSRIs). One TAU-patient got an SSRI medication. All patients in the TAU group had a (recurrent) depressive disorder according to ICD-10 (six in the BLT group). Two patients under BLT were diagnosed with a combined disorder of conduct and emotions and one patient had a post-traumatic stress disorder. All pre-EEGs were recorded 15.42 (*SD* = 9.75) days after admission [*t* (17) = –1.787; *p* = 0.092].

Baseline measures of vigilance classification stages A1, B23, and mean vigilance were not equivalent between the groups. Although *t*-tests did not show a significant difference between the groups [A1: *T*(17) = 1.983;*p* = 0.057; B23: *T*(17) = –1.639; *p* = 0.12; mean vigilance: *T*(17) = 1.727; *p* = 0.102], the confidence intervals of the effect sizes laid not inside the boundaries of the SESOI, even though the SESOI was determined by the small sample size and was quite large [CI of Cohen’s d, A1(-0.29 1.88); B23(-1.68.19); and mean vigilance (-0.16 1.72)]. Therefore, we decided to focus on the effect of time and time by group interaction effects but not on the group effect.

### Level of Vigilance

Of all paired *t*-tests undertaken for the different vigilance levels, only the test on B23-classification revealed an effect of time for the BLT group but not the TAU group. The percentage of B23 classification was lower (pre = 43%, post = 34%), meaning that patients who received BLT got more aroused. In a repeated-measures ANOVA, the group*time interaction was not significant (see Table 1). Looking at Figure 1, one recognizes a higher percentage of A1 classification after treatment for both groups (decreased vigilance in the post-intervention measure, see Table 2 and Figure 1).

**TABLE 2:** Effect of time on EEG vigilance level.

	Paired *t*-test					
	
		Paired pre-pos				
		
Group	Measure	Mean	*SD*	*t*(16)	*p*	Cohen’s d
*BLT*	*A1*	–0.069	0.128	–1.621	0.144	–0.540
	*B23*	0.090	0.079	3.430	**0.009**	1.143
	*Mean vigilance*	–0.489	0.498	–2.944	**0.019**	–0.981
*TAU*	*A1*	–0.043	0.212	–0.602	0.564	–0.201
	*B23*	0.046	0.298	0.460	0.658	0.153
	*Mean vigilance*	–0.237	1.067	–0.667	0.524	–0.222

**ANOVA**						
	**Time**			Time*group		
		
**Measure**	**F**	**p**	**Cohen’s d**	**F**	**p**	**Cohen’s d**

*A1*	1.830	0.195	0.677	0.104	0.751	0.155
*B23*	1.742	0.205	0.659	0.185	0.673	0.211
*Mean vigilance*	1.185	0.083	0.924	0.410	0.531	0.320

**Descriptive**						
	**A1**		**B23**		**Mean vigilance**	
			
	**Mean**	**SD**	**Mean**	**SD**	**Mean**	**SD**

**BLT**						
*Pre*	0.448	0.338	0.435	0.359	4.039	1.302
*Pos*	0.517	0.357	0.345	0.316	4.528	1.228
**TAU**						
*Pre*	0.193	0.195	0.664	0.244	3.169	0.875
*Pos*	0.246	0.196	0.610	0.258	3.445	1.160

*BLT, Bright Light Therapy; TAU, Treatment As Usual; SD, standard deviation; 16, degrees of freedom; significant pe-post differences are shown in bold.*

The same picture was observed for the mean vigilance classification: BLT showed a significant effect in paired *t*-tests [*T* (8) = 2,944; *p* = 0.019] indicating higher mean vigilance classification after BLT (pre = 4.04, post = 4.53) but there was no group*time interaction in the repeated measures ANOVA [*F*(1, 16) = 0.41, *p* = 0.53].

### Vigilance Stability

There was no effect of time (BLT: Z = –0.535; *p* = 0.593; *r* = 0.12; TAU: Z = –1.131; *p* = 0.258; *r* = 0.26) and no effect of group [χ^2^(4) = 4.958, *p* = 0.292, w = 0.54] on pre-post difference in vigilance stability.

### Electroencephalogram Vigilance and Beck Depression Inventory-II Association

For the BDI-II sum score, we found a negative correlation with vigilance stage B23 (*r* = –0.557; *p* = 0.016) and a positive correlation with mean vigilance (*r* = 0.528; *p* = 0.024) only at follow up, indicating that severe depressive symptoms are associated with higher brain arousal level. At baseline, there was no correlation between vigilance measures and the BDI-II sum score. Additionally, we found no correlation between changes in vigilance and changes in BD II score, nor with baseline BDI-II sum score.

## Discussion

In this study, we observed a statistically significant increase in vigilance in depressive adolescent patients after 10 days of BLT. Some increase in EEG-vigilance was observed for the TAU group as well, although to a lesser extent and not reaching statistical significance. However, this vigilance increase after BLT was neither associated with a change in the BDI-II sum score nor with the BDI-II sum score at baseline. This finding indicates that BLT might have an additional and immediate effect on vigilance, which is probably not related to upregulated brain arousal in depression with symptoms, such as inner tension or prolonged sleep latency (14). Instead, these results are more in line with the improvement of sustained attention and sleepiness reported for sleep-deprived young adults after BLT (23). The normalization effect of the antidepressant intervention on an increased level of brain arousal and EEG-vigilance in adult depressive patients (14) was not observed, which might be due to the short period of only 2 weeks between baseline and follow-up. Add-on effects of BLT probably emerge later in the treatment timeline. Nevertheless, we found a positive correlation of the BDI-II sum score with the B23 vigilance level, indicating that severe depressive symptoms are associated with higher brain arousal levels, as was described for adults (22).

As a limitation, it should be mentioned that treatment effects may depend on the baseline level of vigilance. In this study, patients were not assigned as matches in vigilance, which produced somewhat higher arousal in the BLT group before treatment. Further limitations are the lack of treatment response data in terms of follow-up measures of symptoms and EEG vigilance.

Nevertheless, the chosen markers proved to be reliable and valid indicators for neurobiological changes probably associated with depression and its treatment. Future studies with larger samples and longer follow-up times are necessary to replicate the presented results.

## Data Availability Statement

The raw data supporting the conclusions of this article will be made available by the authors, without undue reservation.

## Ethics Statement

The studies involving human participants were reviewed and approved by Ethics Committee of the Ruhr University Bochum. Written informed consent to participate in this study was provided by the participants’ legal guardian/next of kin.

## Author Contributions

CB analyzed the data and drafted the manuscript for intellectual content. SG acquired the data. SG, AD, LJ, JS, and MK revised the manuscript for intellecutal content. FM revised the manuscript for intellectual content and drafted procedure and study sample description of methods part. OR helped in conceptualizing the study and revised the manuscript for intellectual content. TL and MH designed the study, drafted main part of the introduction, and revised the manuscript for intellectual content. All authors contributed to the article and approved the submitted version.

## Conflict of Interest

The authors declare that the research was conducted in the absence of any commercial or financial relationships that could be construed as a potential conflict of interest.

## Publisher’s Note

All claims expressed in this article are solely those of the authors and do not necessarily represent those of their affiliated organizations, or those of the publisher, the editors and the reviewers. Any product that may be evaluated in this article, or claim that may be made by its manufacturer, is not guaranteed or endorsed by the publisher.

## Footnotes

1
https://research.uni-leipzig.de/vigall/
